# Symmetric dimethylarginine concentrations in dogs with International Renal Interest Society stage 4 chronic kidney disease undergoing intermittent hemodialysis

**DOI:** 10.1111/jvim.15612

**Published:** 2019-09-12

**Authors:** André Nanny Vieira Le Sueur, Silvano Salgueiro Geraldes, Alessandra Melchert, Regina Kiomi Takahira, Michael Coyne, Rachel Murphy, Donald Szlosek, Priscylla Tatiana Chalfun Guimarães‐Okamoto

**Affiliations:** ^1^ Department of Veterinary Clinics, School of Veterinary Medicine and Animal Science São Paulo State University—UNESP São Paulo Brazil; ^2^ IDEXX Laboratories Inc Westbrook Maine

**Keywords:** canine, dialysis, glomerular filtration rate, SDMA renal biomarker

## Abstract

**Background:**

Symmetric dimethylarginine (SDMA) is a methylated arginine derived from intranuclear methylation of l‐arginine by protein‐arginine methyltransferase and released into circulation after proteolysis. It is primarily eliminated by renal excretion, and its concentration is highly correlated with glomerular filtration rate (GFR) in animals and humans and is an earlier indicator of kidney dysfunction than serum creatinine concentration (sCr).

**Objectives:**

To evaluate and quantify the effects of IV fluid therapy (IF) or intermittent hemodialysis (IH) on renal function in a randomized group of dogs previously diagnosed with International Renal Interest Society (IRIS) stage 4 chronic kidney disease (CKD).

**Animals:**

Twenty‐four client‐owned dogs with naturally occurring CKD.

**Methods:**

Serum from 14 dogs treated by IH and 10 dogs treated with IF was submitted for measurement of sCr and SDMA. Dogs in each treatment group received up to 5 treatment sessions, administered 48 hours apart.

**Results:**

Significant differences (*P* ≤ .05) were seen between treatment groups, but dogs from the IH group were the most affected based on SDMA (*P* < .001), sCr (*P* < .001), and blood urea (*P* < .001) concentrations. Furthermore, for each 10% increase in urea reduction ratio, there was a 6.2 μg/dL decrease in SDMA (*P* = .002).

**Conclusions and Clinical Importance:**

Although SDMA is dialyzable biomarker and despite its removal by IH, SDMA correlates better with renal function than does sCr in dogs with CKD undergoing IF and IH.

**Abbreviations**
ADMA: asymmetric dimethylarginine
AKI: acute kidney injury
CKD: chronic kidney disease
GFR: glomerular filtration rate
IDH: intradialytic hypotension
IF: IV fluid therapy
IH: intermittent hemodialysis
IRIS: International Renal Interest Society
RRT: renal replacement treatment
SBP: systolic blood pressure
sCr: serum creatinine
SDMA: symmetric dimethylarginine
UF: ultrafiltration
URR: urea reduction ratio

## 1 INTRODUCTION

Renal disease is highly prevalent in companion animal clinical practice and can be associated with poor prognosis, especially when diagnosed late in the course of the disease.^1^ Chronic kidney disease (CKD) can be a combination of single or multiple insults that, when incompletely repaired, lead to irreversible damage to the structure and function of the parenchyma in one or both kidneys.^2^

Recently, the International Renal Interest Society (IRIS) recommended using serum creatinine concentration (sCr) to diagnose and stage CKD, acknowledging symmetric dimethylarginine (SDMA) as an adjunct for both diagnosis and staging.^3^ It is an amino acid originating from protein degradation through arginine methylation and is a biomarker that is freely filtered by the kidneys.^1^, ^2^, ^4^, ^5^ This biomarker is similar in size to creatinine, and its serum concentration correlates well with glomerular filtration rate (GFR) and with sCr in humans, dogs, cats, rats, and mice.^4^ In animals, SDMA has been shown to have greater sensitivity, and its concentration increases with an average of 40% decrease in GFR as compared to sCr, which only increase when approximately 75% of kidney function is impaired.^5^, ^6^, ^7^

Intermittent hemodialysis (IH) is a renal replacement treatment (RRT) widely prescribed throughout veterinary practice. Dialysis is indicated for animals undergoing severe uremic crisis because of acute kidney injury (AKI), or CKD, or in animals with fluid overload or refractory cardiogenic pulmonary edema, acid‐base, and electrolyte imbalances, and for the removal of certain drugs and toxins.^8^ Intermittent hemodialysis is prescribed in animals with CKD to correct comorbidities generated by severe uremia and to improve quality of life for those not responding to conservative clinical treatment.^9^

Studies in people have shown that SDMA is a dialyzable molecule similar to creatinine, and its concentration was not influenced by different types of dialyzers.^10^, ^11^, ^12^ Likewise, other studies suggest that serum SDMA concentrations progressively increase according to the stages of CKD and especially in those receiving RRT.^13^, ^14^, ^15^ Thus, our main objective was to evaluate the effects of IH on the serum concentrations of SDMA in dogs previously diagnosed with IRIS stage 4 CKD.

## 2 MATERIALS AND METHODS

This study was approved by the Ethics Committee on Animal Use (CEUA) of the School of Veterinary Medicine and Animal Science—São Paulo State University (UNESP) Botucatu (protocol 15/2016).

### 2.1 Animals and study design

Twenty‐four client‐owned dogs of both sexes, and various ages and breeds were prospectively selected from the patient population of the Nephrology and Urology Small Animal Service of the Teaching Hospital of the School of Veterinary Medicine and Animal Science, São Paulo State University. All dogs were previously diagnosed with IRIS stage 4 CKD.^3^

Dogs were evaluated during the 30‐day period before inclusion into the study. Laboratory evaluation including CBC, urinalysis, urinary protein:creatinine ratio, venous blood gas analysis, vector‐borne disease PCR (if needed), and serum biochemistry were performed weekly. All patients also had thoracic radiography and abdominal ultrasonography performed. Dogs that developed AKI, shock, sepsis or were being treated for pancreatitis, autoimmune diseases, congestive heart failure, neoplasia, coagulation disorders, infectious diseases, nephrolithiasis, or had a previous diagnosis of familial or congenital renal disease were excluded from the study.

Dogs were divided into 2 groups: the IH group (n = 14) and the IV fluid therapy (IF) group (n = 10). Patient selection was based on the pet owner's time availability and location of residence. After randomization, any dog assigned to the IH group of which the owner subsequently refused IH for personal reasons was moved to the IF group. Dogs in the IH group underwent up to 5 treatment sessions 2‐3 times week at a 48‐hour minimum interval. Dogs in the IF group received treatment as needed for CKD either at the teaching hospital or at home by the pet owners. Dogs in both groups were evaluated every 48 hours during the 2‐week study.

### 2.2 CKD clinical treatment

#### 2.2.1 IV fluid therapy

Dogs received an isotonic polyionic replacement crystalloid such as lactated Ringer's solution. The fluid rate was based on the estimation of hydration of the patient (body weight × estimated dehydration deficit as a percentage) plus a maintenance rate (2‐6 mL/kg/h) and a volume to account for ongoing losses (polyuria, vomiting, diarrhea) if present.^16^ During hospital operation (12 hours), a constant rate infusion (CRI) was utilized, but most of the patients also received SC fluids from the pet owners at home on days when absent from the study. Systolic blood pressure (SBP) was monitored at each visit by Doppler (Doppler Vascular Parks ) throughout the study period.

All pharmacological treatments also were administered at recommended dosages.^17^, ^18^ Proton pump inhibitors and antiemetics such as omeprazole (Omeprazol, EMS S.A.; 0.5‐1 mg/kg PO q12‐24h), ondansetron (Vonau, BIOLABFARMA; 0.1 mg/kg PO q8h), and maropitant citrate (Cerenia, Zoetis; 1 mg/kg SC q24h for 5 days) were prescribed for gastroprotection and to control nausea and vomiting if needed. Human‐recombinant erythropoietin (Eritromax, Blau Farmacêutica S.A.; 100 UI/kg SC q48h) was used to control nonregenerative anemia when hematocrit was ≤15%, and iron supplementation was administered as adjuvant treatment along with erythropoietin treatment. All hypertensive and proteinuric dogs received an angiotensin‐converting‐enzyme inhibitor as monotherapy or combined with a calcium channel blocker if needed. In this prospective study, all dogs were proteinuric. Lastly, hyperphosphatemia was managed by using aluminum hydroxide (Hidróxido de Alumínio, Sanofi, 90 mg/kg PO q24h) and a commercial renal diet (Royal Canin Renal Diet).^19^

#### 2.2.2 Intermittent hemodialysis

All dialysis sessions were performed using a 4008S Fresenius machine (Fresenius Medical Care). Selected dogs were catheterized with an 11 French double‐lumen catheter (VetMedical) in the right jugular vein using the Seldinger technique.^20^ Subsequently, a radiographic examination was performed to ensure correct location of the catheter in the patient. For dialysis prescription and adequacy, an algorithm based on the urea reduction ratio (URR) was used after treatments. Because of inexperience with dialysis adequacy, an empirical blood flow^8^ (Qb) was set according to serum urea concentrations, and hemodialyzers (Hemoflow, Fresenius Medical Care) were chosen according to the patient's body weight based on veterinary literature.^8^, ^21^ All lines and dialyzers received a priming solution of sterile saline.

Prescriptions for IH were set with a Qb between 2 and 5 mL/kg/min; ultrafiltration (UF) rates were kept constant between 5 and 10 mL/kg/h because of the priming solution of 120 mL, and subsequently because of boluses of crystalloids used as a consequence of poor catheter performance in 3 dogs. Treatment time for IH was 60 to 180 minutes. A bicarbonate solution (BiBag, Fresenius Medical Care) was added to the dialysate solution and kept at a constant flow rate (Qd) of 500 mL/min during all sessions. Anticoagulation was achieved using unfractionated heparin (Liquemine, Roche) at an initial IV dosage of 50 UI/kg. An activated clotting time machine (MCA 2000—Fundação Adib Jatene) was used to measure the anticoagulant effect hourly, and additional boluses of heparin were administered if necessary.^22^ Systolic blood pressure also was monitored by Doppler (Parks Medical Electronics—Ultrasonic Doppler Flow Detector 811‐B) every 30 minutes throughout the dialysis treatment.^23^

#### 2.2.3 Sample collection and analytical methods

In the IF group, blood was drawn via jugular venipuncture at baseline (pre‐IF) and 30 minutes after fluid therapy (post‐IF) for each in‐hospital session. In the IH group, baseline samples were collected from the double‐lumen catheter (pre‐IH) and 60 minutes after dialytic treatment (post‐IH) to avoid recirculation effect. After collection, blood was transferred into serum tubes and centrifuged at 3000*g* for 10 minutes. Serum then was decanted, stored in sealed aliquots, frozen, and stored at −80°C for subsequent biochemical analysis. Because SDMA was only available in the United States during this study, kidney function was measured only by sCr at baseline and compared to SDMA concentrations at the end of the study after samples had been sent as a batch to the laboratory in the United States.

Symmetric dimethylarginine and sCr were measured in each sample, in duplicate, at a commercial laboratory. Symmetric dimethylarginine was determined using a commercially available high‐throughput immunoassay (IDEXX SDMA Test; IDEXX Laboratories Inc, One IDEXX Drive, Westbrook, Maine). Serum creatinine concentration was determined using a colorimetric method, Jaffe's reaction using picrate at alkaline pH^24^ (Beckman Coulter, Inc, Brea, California). Both assays were performed according to the manufacturers' recommendations. The dynamic range of the 2 assays was 0‐100 μg/dL and 0.045‐50 mg/dL for SDMA and sCr, respectively.

### 2.3 Statistical analysis

Although some animals had >5 treatment sessions, only results up to and including 5 treatment sessions were included according to the study protocol. A Wilcoxon Sign Rank Sum test was used to determine if treatment groups were similar. A linear mixed effect model was used to evaluate the differences between IH and IF on measures of SDMA, sCr, phosphorus, blood urea, and serum protein concentrations. The model accounted for the random effects of patient and the fixed effects of age, breed, sex, body weight, neuter status, and sample time (pre‐ and post‐treatment). Differences in treatment groups were evaluated and reported as maximum likelihood estimates.

To evaluate how SDMA and sCr behaved in combination with urea as a response to IH and IF, an interaction term for treatment and urea was added to the respective models. Likewise, a linear mixed model was performed accounting for the random effects of patient and fixed effects to test if a difference in SDMA was associated with a difference in SBP. Values were taken before and after treatment by a day and compared.

Finally, a linear mixed model was performed accounting for the random effects of patient and the fixed effects to test if a change in SDMA or sCr was associated with URR. The differences in SDMA and sCr before and after treatment were compared to URR and reported as maximum likelihood estimates. All linear mixed models were tested by comparing the values of the likelihood functions of the reference model (the full model) against the reincluded model (the nested model without the reincluded variables) using the likelihood ratio test. All statistical analysis was performed using R version 3.3.3, and the linear mixed models were done using the “lme4” package^25^ that included the random effects of “dog,” “sample,” and “duplicates.” For all tests, *P < *.05 was considered statistically significant.

## 3 RESULTS

The demographic and clinical characteristics of the study population are listed in Table 1. Among the 63 hemodialysis sessions performed, 1 dog developed dialysis disequilibrium syndrome and intradialytic hypotension (IDH) occurred in 2 dogs. Furthermore, increased flow resistance occurred in 3 dogs from the IH group because of poor catheter performance.

**Table 1:** Demographic and clinical characteristics of the study population

	Group IH (n = 14)	Group IF (n = 10)
Age, y	8 ± 3	11 ± 4
Sex		
Male intact	5	5
Male neuter	3	3
Female intact	2	0
Female neuter	4	2
History of hypertension (yes/no)	5/9	8/2
Mean systolic blood pressure (mm Hg)	150.9 ± 16.6	157.0 ± 5766
History of proteinuria (yes/no)	14/0	10/0
Median UPC (IQR)	1.8 (1.5‐2.5)	2.4 (1.9‐3.2)
Median WBC (IQR)	8.8 (7.05‐10.58)	7.9 (6.75‐10.53)
History of use of ACEis/CCB	2/3	4/4
History of the use of combined treatment (ACEi + CCB) (yes/no)	5/9	2/8
History of the use of phosphate binders (yes/no)	14/0	10/0
History of the use of EPO (yes/no)	4/10	1/9
History of the use of gastroprotectants (yes/no)	14/0	10/0

Abbreviations: ACEi, angiotensin‐converting enzyme inhibitor; CCB, calcium channel blocker; EPO, erythropoietin; IF, IV fluid therapy; IH, intermittent hemodialysis; IQR, interquartile range; UPC, urinary protein:creatinine ratio.

**Figure 1:**
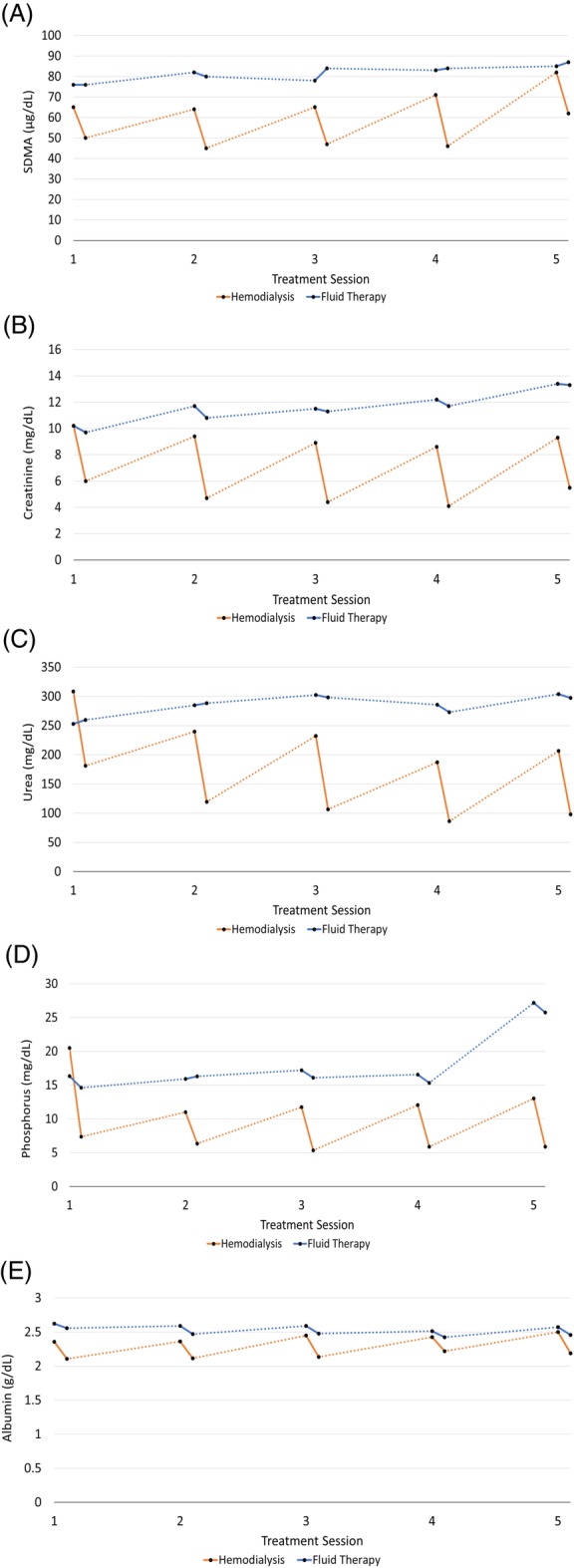
Mean analyte measures pre‐ and post‐treatment by treatment session. A, Symmetric dimethylarginine. B, Serum creatinine. C, Urea. D, Phosphorous. E, Serum albumin

Pre‐ and post‐treatment sCr and SDMA are listed for each treatment group in Tables A1 and A2). Before the start of treatment, both groups were observed to have similar blood chemistry results and body weight (Table 2). The changes in median values for each analyte above over time are shown in Figure 1.

**Table 2:** Blood chemistry and body weight measures before first treatment by treatment group

	Hemodialysis mean (95% CI)	Fluid therapy, mean (95% CI)	*P*‐value^a^
SDMA (μg/dL)	68.3 (24.4‐90.1)	77.3 (36.0‐112.0)	.30
Creatinine (mg/dL)	10.4 (1.6‐12.2)	10.4 (4.2‐16.2)	.98
Albumin (g/dL)	2.2 (1.8‐2.6)	2.6 (2.0‐3.2)	.18
Urea (mg/dL)	225.6 (97.4‐353.8)	232.3 (124.8‐339.7)	.27
Phosphorus (mg/dL)	10.4 (2.1‐18.7)	14.6 (9.0‐20.2)	.83
Body weight (kg)	19.0 (7.6‐30.5)	22.2 (8.8‐35.6)	.77

Abbreviations: CI, confidence interval; SDMA, symmetric dimethylarginine.

a Wilcoxon signed rank sum test.

Values of the linear mixed effect model accounting for pre‐ and post‐treatment and day of session were used to evaluate differences in the maximum likelihood estimates between both treatments and are found in Table 3. Significant differences in the maximum likelihood estimate of the mixed effect models (*P <* .001) were seen between treatment groups for SDMA, sCr, BUN, serum albumin, and phosphorus concentrations, with lower values in the IH group. To test how SDMA and sCr behaved in combination with BUN as a response to IH and IF, an interaction term for treatment and urea was added to the respective models. The interaction of urea and treatment in the SDMA model was not significant (Table 3). The interaction term in the sCr model was statistically significant (Table 3).

**Table 3:** Linear mixed model with biomarkers as dependent variables^a^

Reincluded variable	Dependent variable	Shift in dependent variable	Standard error	*P*‐value
Treatment (IF/IH)	SDMA	−26.13	5.087	<.001
Treatment (IF/IH)	Creatinine	−3.75	0.667	<.001
Treatment (IF/IH)	Urea	−116.50	16.712	<.001
Treatment (IF/IH)	Albumin	−0.25	0.060	.10
Treatment (IF/IH)	Phosphorous	−1.300	2.442	<.001
Treatment (IF/IH)^b^	ΔSDMA	0.347	0.0546	<.001
Treatment (IF/IH)^b^	ΔCreatinine	4.582	0.422	<.001
Treatment (IF/IH)× urea	SDMA	0.024	0.046	.65
Treatment (IF/IH)× urea	Creatinine	0.017	0.005	<.001
URR (%)	SDMA	−0.621	0.01	.002

Random effects: Patient_ID.

Abbreviations: IF, IV fluid therapy; IH, intermittent hemodialysis; SDMA, symmetric dimethylarginine; URR, urea reduction ratio.

a Fixed effects: sample time (pre/post), treatment session, breed, age, sex, body weight, neuter status.

b The difference in SDMA and creatinine values were taken pre‐/post‐treatment and compared to URR.

**Figure 2:**
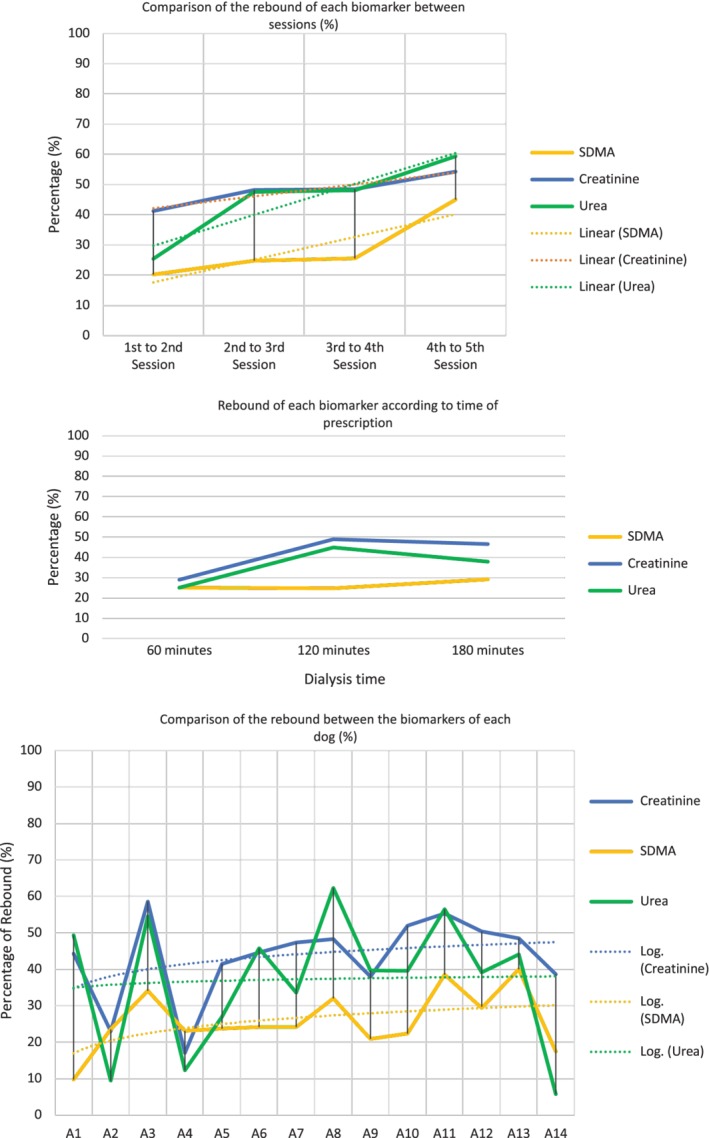
The median 48‐hour rebound effect of symmetric dimethylarginine compared with serum urea and serum creatinine rebounds between sessions, according to time of prescription, and compared individually

Symmetric dimethylarginine was shown to be a dialyzable molecule by a significant association between URR and SDMA. As a result of each 10% increase in URR, a 6.2 μg/dL decrease in SDMA was observed (*P =* .002; Table 3). Between treatments, SDMA had a clearance of 28% by dialytic treatment. Furthermore, SDMA concentrations demonstrated a median 48‐hour rebound of 25% between sessions and 24% in each dog over the duration of the study. Because the duration of treatment also influences the rebound effect, SDMA concentrations showed a 48‐hour rebound effect of 25% in sessions that lasted 60 and 120 minutes and a 29% rebound in those that underwent 180 minutes of treatment. The rebound of SDMA was lesser and steadier when compared to the rebounds of BUN and sCr (Figure 2). Although a significant association between URR and SDMA was shown, testing the difference between SDMA and sCr before and after treatment by URR showed only a weak association (Table 3). Symmetric dimethylarginine weakly increased URR by 0.347 ± 0.0546 (*r* ^2^ = .229, *P <* .001) and sCr increased URR by 4.582 ± 0.422 (*r* ^2^ = .270, *P <* .001). Additionally, no significant association was observed between SBP and SDMA concentrations for either treatment (*r* ^2^ = .04, *P* = .26).

## 4 DISCUSSION

We confirmed the effectiveness of IH in the removal of urea, creatinine, and phosphorus from serum. Intermittent hemodialysis also was effective for SDMA clearance but limited as a long‐term treatment modality in dogs with IRIS CKD stage 4, similar to humans who underwent IH for end‐stage renal disease.^10^, ^11^, ^12^, ^13^, ^26^, ^27^, ^28^, ^29^ In 1 study of humans, the marked increase in SDMA concentration was approximately 3.5‐fold higher in early stage CKD patients, whereas it was more than 7‐fold higher both in peritoneal dialysis and hemodialysis patients in end‐stage CKD when compared to healthy controls.^14^

Another study comparing dialytic clearance between SDMA and asymmetric dimethylarginine (ADMA) in human patients in dialysis sessions demonstrated extraction of 40% and 28%, respectively, for both markers, almost a 2‐fold difference between both markers of similar molecular weight.^27^, ^30^ Conversely, in another study that evaluated SDMA extraction in human patients with AKI undergoing continuous RRT, SDMA had a lower clearance of 9% during a 12‐hour interdialytic interval.^31^ Interestingly, in another study of humans that evaluated dimethylarginine clearances using membranes of different permeability and biocompatibility, the group that underwent hemofiltration using a high‐flux membrane with an infusion volume of 7.5 L experienced more efficiency than the hemodialysis group with the respect to the clearance of SDMA.^32^ Hence, our prospective study demonstrated that SDMA concentration had a clearance of 28% and a decrease of 6.2 μg/dL for each 10% increase in URR per session.

Despite SDMA and sCr having a small association with URR (Table 3), we suggest that IH limits SDMA extraction because of its different molecular weight, by its increased body distribution volume (because of progressive loss of kidney function), and its accumulation in the high‐density lipoprotein fraction in patients with CKD, as previously reported in studies of humans.^13^, ^27^, ^31^ Furthermore, IH also has been reported as a pro‐inflammatory and catabolic treatment,^12^, ^14^ and this RRT modality also can trigger methylated arginine generation, associated with an increased volume of distribution of this biomarker.^11^, ^27^, ^32^, ^33^ Therefore, urea kinetics is not representative for the removal of other solutes such as methylarginines, and consequently, hemodialysis is not suitable for a long‐lasting removal of methylarginines from the bloodstream.^27^, ^33^, ^34^, ^35^

The IF group had higher serum concentrations of SDMA throughout the study, whereas in the IH group, serum SDMA concentrations progressively increased (27% compared to a 13.6% increase of the IF group). Intermittent hemodialysis was demonstrated to be a better and more efficient treatment modality for sCr extraction (*P* < .001; Table 3), as described previously.^8^, ^9^, ^10^ In our study, IH achieved a 9% decrease in sCr concentrations, whereas in the IF group, sCr concentrations increased 31%. As an endogenous marker of renal function, SDMA is closely related to GFR,^36^, ^37^, ^38^, ^39^, ^40^ less affected by IH clerance,^12^, ^13^, ^14^, ^24^, ^28^, ^30^, ^31^ and less affected by hydration status, providing a better clinical representation of kidney function and disease progression than sCr in IH‐treated dogs.

The main advantages of IH are the reduction of water‐soluble uremic toxins and be a superior treatment for the control of CKD comorbidities, such as fluid, electrolyte, and acid‐base imbalances that are corrected more effectively by IH than IF.^8^, ^9^ Despite serum phosphorus concentrations, being significantly changed in the dialysis group (Figure 1D and Table 3), the lowering of phosphorus to recommended serum concentrations (<6.0 mg/dL)^19^ was not fully achieved because of the short evaluation period, slow rates of phosphate transfer from the intracellular to the extracellular pool or possibly to the rapid phosphorus rebound effect that occurs immediately after the dialytic treatment.^41^

The interdialytic rebound effect was described not only for phosphorus concentrations but for all solutes (eg, SDMA, urea, creatinine), markers commonly utilized to evaluate the reduction rate efficacy of the dialytic treatment.^21^ All dogs in our prospective study were diagnosed and included at IRIS Stage 4 CKD (end‐stage). Therefore, an interdialytic rebound effect was observed as “peaks” in serum concentrations of SDMA, creatinine, urea, and phosphorus (Figures 1A‐D), as previously described in studies of humans.^14^, ^27^, ^36^, ^37^, ^38^, ^40^, ^41^

Measurement of the rebound effect can be determined by different methods and formulas, and the major component of the interdialytic rebound effect is a result of solute transfer between compartments, commonly measured using urea or creatinine because of their molecular size and distribution in the body.^8^, ^21^, ^42^ Notwithstanding, we also evaluated the rebound effect of SDMA in each patient, between sessions and according to the duration of each treatment prescription, and then compared its rebound with that of urea and creatinine.

Our study demonstrated a 48‐hour rebound of 25% in SDMA concentration based on the duration of prescription and time between each session. Likewise, a rebound of 24% in SDMA was seen over the 15 days of study. Symmetric dimethylarginine had a smaller and steadier post‐dialytic rebound when compared to urea and creatinine, even in shorter sessions with higher URRs.

Dialysis disequilibrium syndrome is induced by rapid changes in blood composition and osmolality as a consequence of intense and rapid dialysis clearance, resulting in a lower plasma osmolality, and causing cerebral edema.^8^ Signs may include agitation, disorientation, seizures, coma, or death.^9^, ^21^ We observed agitation and vocalization in 1 dog, which was treated supportively using mannitol (500 mg/kg/ IV) and increased dialysate sodium modulation by the dialysis machine. Intradialytic hypotension was seen in 2 dogs, and both were treated using IV colloid fluid administration, lowering the dialysate temperature and by deactivation of UF, all of which are recommended techniques.^8^, ^21^

Although IDH is related to larger dialytic decreases in SDMA concentrations during hemodialysis,^26^ in our study, IDH was reported in dogs with lower decreases in SDMA (27%), and interestingly, IDH occurred in both dogs that were treated with angiotensin‐converting enzyme inhibitors in association with calcium channel blockers for proteinuria and hypertension. Three dogs that experienced poor catheter performance because of short catheter length demonstrated a decrease in both sensors of transmembrane pressure and venous pressure, respectively. Therefore, all dogs received boluses of crystalloid solution within the sessions in association with UF to avoid fluid overload until catheter replacement.

During our study, kidney function was evaluated during both treatments exclusively by the magnitude of azotemia. Subsequently, when serum SDMA concentrations were analyzed, disease progression seemed clear, something that could not be determined by sCr alone, especially for the IH group for which BUN and sCr were unreliable because of their extraction. In our study, SDMA was shown to be a more stable biomarker compared to creatinine in dogs that underwent IH, being less influenced by clearance and by the post‐dialytic rebound effect.

We hypothesize that the increase in SDMA concentration in dogs that underwent IH can be related to many factors, such as its different molecular weight and its distinct dialytic clearance. Moreover, the accumulation of SDMA in the high‐density lipoprotein fraction, increased body distribution of methylarginines related to the progressive worsening of kidney function, and the impact on SDMA concentrations of protein catabolism are additional facts to consider.^12^, ^14^

Our study had a number of limitations. An assessment of body mass and muscle mass was not conducted. In our clinical evaluation, we divided the nutrition scoring system into visual categories and body weight, neither ultrasound nor computed tomography was utilized to assess muscle mass in these animals. Additionally, although there is a correlation between sCr and lean body mass in dogs,^43^ serum concentrations of SDMA also may have been impacted with malnourishment, as previously determined in human patients undergoing IH.^12^ Thus, an objective assessment of body condition score and muscle mass would have allowed a better estimation for both markers affected by protein catabolism and urea generation. The time frame of our study was too short to show progressive changes in SDMA versus daily variation in a long duration dialysis treatment. We could not correlate SDMA with a gold standard GFR evaluation (eg, inulin or iohexol clearance) because of the limited funding and lack of test availability in Brazil. Only SDMA was measured and not evaluated in relationship to nitrous oxide, l‐arginine, and its methylated forms (ADMA, dimethylarginine dimethylaminohydrolase, and NG‐monomethyl‐l‐arginine). In human patients, these compounds potentially are involved in the pathophysiology of endothelial dysfunction, oxidative stress, nutrition, apoptosis, atherosclerosis, uremia, autophagy, inflammation, and impaired immunological function which can worsen the kidney function.^28^, ^29^, ^30^, ^34^, ^35^, ^36^, ^37^, ^44^, ^45^, ^46^, ^47^ Lastly, we were unable to determine if a silent and sustained AKI process could have been present and undetectable by routine conventional biochemical laboratory assays. Thus, use of novel markers of active AKI may facilitate early recognition, monitoring, and be a better prognostic indicator in dogs with CKD, especially those on RRT.

In conclusion, although SDMA is a dialyzable biomarker, its clearance depends mainly on renal function, and despite multifactorial and complex mechanisms that still are not fully elucidated, in our study SDMA was found to be a promising biomarker for monitoring dogs with CKD undergoing both IH and IF. This biomarker also was able to detect progression of CKD, especially in those animals having their other biomarkers cleared by the dialytic treatment, and SDMA also was less influenced by the post‐dialytic rebound effect. Furthermore, IH proved to be a superior and highly effective therapeutic modality when compared to IF in the removal of uremic solutes and the correction of fluid and electrolyte imbalances in dogs with IRIS stage 4 CKD. In contrast, IH may not be an effective long‐term therapeutic modality for SDMA removal.

## CONFLICT OF INTEREST DECLARATION

IDEXX Laboratories Inc. has provided SDMA and sCr analysis at no charge as support collaboration for the study. Michael Coyne, Rachel Murphy, and Donald Szlosek are currently employed by IDEXX Laboratories, Inc. Priscylla Tatiana Chalfun Guimarães Okamoto, Alessandra Melchert, Regina Kiomi Takahira are currently researchers of the São Paulo State University, Andre Nanny Vieira Le Suer and Silvano Salgueiro Geraldes are currently PhD student of the São Paulo State University; Priscylla Tatiana Chalfun Guimarães Okamoto, Alessandra Melchert, and Regina Kiomi Takahira are also professor employees in the same institution.

## OFF‐LABEL ANTIMICROBIAL DECLARATION

Authors declare no off‐label use of antimicrobials.

## INSTITUTIONAL ANIMAL CARE AND USE COMMITTEE (IACUC) OR OTHER APPROVAL DECLARATION

This study was approved by the Ethics Committee on Animal Use (CEUA) of the School of Veterinary Medicine and Animal Science, São Paulo State University, UNESP, Botucatu—SP protocol no 15/2016, CEUA.

## Supporting information


**Appendix S1:** Supporting Information
