# Supplementary material for: Symmetric dimethylarginine concentrations in dogs with International Renal Interest Society stage 4 chronic kidney disease undergoing intermittent hemodialysis
Source: J Vet Intern Med. 2019 Sep 12;33(6):2635–43. doi: 10.1111/jvim.15612 (PMC6872610; doi:10.1111/jvim.15612)
Supplement: Supplementary file 1 — Appendix S1: Supporting Information [file JVIM-33-2635-s001.pdf]

Supplementary Tables

Table A1.A– Online appendix: Serum creatinine (mg/dL) Pre- and Post-Values; number (*N*), mean, median and standard deviation (SD) of dogs undergoing intermittent hemodialysis

Dogs	*Sessions*													
	1		2		3		4		5		6		7	
	Pre	Post	Pre	Post	Pre	Post	Pre	Post	Pre	Post	Pre	Post	Pre	Post
1H	10.1	5.4	9.7	3.0										
2H	9.8	5.7	7.4	2.9										
3H	12.2	2.9	5.8	2.4	7.3	2.4								
4H	6.5	5.0	5	3.7	4.2	2.8	3.6	2.7						
5H	10.4	7.0	12.2	5.8	9.9	5.7	9.5	5.2						
6H	7.3	5.0	7.2	3.1	7.2	4.6	8.3	4.1						
7H	6.1	3.7	7.8	5.8	8.6	2.7	7	4.4	7.6	3.0				
8H	7.7	4.1	5.8	2.4	5.3	2.8	5.8	2.7	4.9	1.9				
9H	6.8	4.9	6.9	4.5	8.5	4.8	6.1	3.4	7.3	5.6				
10H	17.6	7.4	12.3	4.5	10.4	4.5	12.1	5.8	11	3.6				
11H	11.5	4.6	10.3	7.0	7.6	3.0	7.5	0.9	7.3	3.8	7.8	5.9		
12H	23.9	17.5	21.4	9.0	17.8	6.8	16.1	6.5	14.8	14.4	13.3	5.2	10.7	3.3
13H	8.9	4.7	8.7	3.9	11.3	5.2	10.1	5.7	14.1	6.6	11	5.4		
14H	10.7	6.4	11.9	8.6	8.9	8.0	8.6	3.5	7.8	5.5	8	5.5	10.2	8.6
N	14	14	14	14	12	12	11	11	8	8	4	4	2	2
Mean	10.2	6.0	9.4	4.7	8.9	4.4	8.6	4.1	9.3	5.5	10.0	5.5	10.5	6.0
Median	10.4	5.0	8.3	4.2	8.5	4.5	8.3	4.1	7.7	4.6	9.5	5.5	10.5	6.0
SD	3.55	3.52	4.22	2.19	3.43	1.77	3.38	1.65	3.56	3.90	2.66	0.29	0.35	3.75

Table A1.B – Online appendix: SDMA (µg /dL) Pre- and Post-Values; number (*N*), mean, median and standard deviation (SD) of dogs undergoing intermittent hemodialysis

Dogs	*Sessions*													
	1		2		3		4		5		6		7	
	Pre	Post	Pre	Post	Pre	Post	Pre	Post	Pre	Post	Pre	Post	Pre	Post
1H	89	74	82	36										
2H	74	52	68	56										
3H	96	40	48	33	68	40								
4H	25	17	30	25	28	20	26	22						
5H	63	58	93	61	80	63	81	63						
6H	57	60	65	44	58	47	78	52						
7H	67	47	50	41	71	46	87	67	69	53				
8H	100	100	90	61	100	69	92	54	100	77				
9H	70	51	61	47	63	56	62	47	81	56				
10H	60	45	55	42	54	50	91	65	84	58				
11H	75	51	83	61	79	39	86	16	98	67	100	83		
12H	30	36	48	27	51	35	47	30	58	55	56	46	69	50
13H	26	21	27	22	43	30	50	36	69	41	65	47		
14H	75	50	93	73	86	69	86	56	100	94	100	97	100	100
N	14	14	14	14	12	12	11	11	8	8	4	4	2	2
Mean	64.6	49.9	63.6	44.6	64.9	46.8	71.2	45.8	82.3	62.4	80.1	68.0	84.3	74.8
Median	68.3	50.0	62.5	42.8	65.0	46.5	80.5	52.0	82.5	56.8	82.3	64.8	84.3	74.8
SD	24.04	20.71	22.07	15.68	19.84	15.45	22.04	17.58	16.19	16.45	23.21	25.71	22.27	35.71

Table A2.A – Online appendix: Serum creatinine (mg/dL) Pre- and Post-Values; number (*N*), mean, median and standard deviation (SD) of dogs submitted to intravenous fluid therapy.

Dogs	*Sessions*															
	1		2		3		4		5		6		7		8	
	Pre	Post	Pre	Post	Pre	Post	Pre	Post	Pre	Post	Pre	Post	Pre	Post	Pre	Post
1F	10.2	8.2	13.7	*												
2F	12.9	12.5	12.2	11.9	13.1	12.6										
3F	6.8	8.3	9.4	9.9	9.9	10.0	9.7	9.9								
4F	7.2	6.6	11.3	10.4	10.7	9.7	9.0	9.1	9.3	10.6						
5F	11.3	10.9	15.0	11.2	13.2	14.8	19.6	18.9	23.8	19.5						
6F	6.6	6.8	6.4	6.0	7.1	9.1	6.4	5.6	5.3	6.0	6.4	5.8	8.4	10.9	9.0	8.5
7F	10.6	10.2	11.0	10.2	10.0	8.7	10.4	11.0	14.5	13.3	10.9	12.6				
8F	15.8	13.1	16.6	15.4	17.1	15.8	22.6	17.8	20.8	23.8	24.2	26.2				
9F	6.0	5.5	5.9	6.5	6.2	6.2	6.3	6.3	6.4	6.5	7.1	8.1				
10F	15.0	15.1	16.0	16.2	16.2	14.6	13.8	15.0	13.6	13.8	12.0	11.6				
N	10	10	10	9	9	9	8	8	7	7	5	5	1	1	1	1
Mean	10.2	9.7	11.7	10.8	11.5	11.3	12.2	11.7	13.4	13.3	12.1	12.9	8.4	10.9	9.0	8.5
Median	10.4	9.2	11.7	10.4	10.7	10.0	10.0	10.5	13.6	13.3	10.9	11.6	8.4	10.9	9.0	8.5
SD	3.55	3.15	3.73	3.44	3.74	3.29	6.04	5.02	7.01	6.51	7.15	7.95				

* Second sample from Case 1F post treatment did not have sufficient volume for analysis

Table A2.B – Online appendix: SDMA (µg /dL) Pre- and Post-Values; number (*N*), mean, median and standard deviation (SD) of dogs submitted to intravenous fluid therapy.

Dogs	*Sessions*															
	1		2		3		4		5		6		7		8	
	Pre	Post	Pre	Post	Pre	Post	Pre	Post	Pre	Post	Pre	Post	Pre	Post	Pre	Post
1F	87	74	100	*												
2F	100	100	100	100	100	100										
3F	51	79	61	72	61	66	87	90								
4F	72	73	97	100	94	83	81	95	60	85						
5F	53	57	90	68	81	100	100	100	100	95						
6F	75	71	52	59	73	69	69	52	75	63	70	71	73	75	64	47
7F	95	96	100	99	74	84	66	83	87	82	53	74				
8F	49	54	74	68	63	84	100	98	100	100	100	100				
9F	80	60	47	53	61	72	64	54	76	83	70	78				
10F	100	100	100	100	100	100	100	100	100	100	100	100				
N	10	10	10	9	9	9	8	8	7	7	5	5	1	1	1	1
Mean	76.0	76.3	81.9	79.6	78.2	84.0	83.2	83.8	85.2	86.6	78.5	84.4	73.0	75.0	64.0	47.0
Median	77.3	73.0	93.3	71.5	73.5	83.5	83.5	92.3	86.5	85.0	70.0	77.5	73.0	75.0	64.0	47.0
SD	19.98	17.33	21.69	19.74	16.25	13.67	15.81	20.00	15.91	13.21	20.89	14.45				

* Second sample from Case 1F post treatment did not have sufficient volume for analysis
